# Efficacy and Side Effects of Combined Capecitabine Plus Intensity Modulated Radiotherapy as an Effective Adjuvant Therapy for Gastric Cancers

**DOI:** 10.22037/ijpr.2019.14622.12542

**Published:** 2020

**Authors:** Jie Fu, Chun-Yan Wang, Chun-Gang Wang, Hong-Ling Li, Xiao-Jing Yang, Yi Sun, Yu-Hui Shao, Li-Hua Zhang, Xin-Miao Yang, Xiu-Long Zhang

**Affiliations:** a *Department of Radiation Oncology, Shanghai Jiao Tong University Affiliated Six People’s Hospital, No 600, Yishan Road, Shanghai 200233, China. *; b *Department of Medical Oncology, Shanghai Pulmonary Hospital, Tongji University, Shanghai 200310, China. *; c *Department of Radiation Oncology, Shanghai Jiao Tong University Affiliated First People’s Hospital, No 100, Haining Road, Shanghai 200233, China.*; 1 *J. F. and C. Y. W. contributed equally to this work*

**Keywords:** Gastric cancer, Intensity modulated radiotherapy (IMRT), Capecitabine, Adjuvant therapy, Side effects

## Abstract

This study aims to evaluate the clinical outcomes and the toxicities associated with intensity modulated radiotherapy (IMRT) administered in combination with capecitabine for gastric cancer. This study was conducted between July 2009 and October 2011, and included 31 patients (23 female and eight male patients; mean age: 57 years old) with pathologically confirmed gastric cancer (pathological staging T3 or T4 or positive lymph node). All patients underwent D2 surgery and adjuvant chemoradiotherapy, followed by combined treatment with IMRT and capecitabine. All patients received follow-up examinations every 3-6 months by physical examination, magnetic resonance imaging (MRI), and assays for tumor markers. The Kaplan-Meier method was used to calculate the rates for locoregional control (LRC) and disease-free survival (DFS). Only two patients could not complete the planned treatment regimen. Patients treated with IMRT and capecitabine tolerated their treatment well, and displayed few significant side effects. The mean follow-up, disease-free survival (DFS) and survival times were 33.0, 27.5, and 32.9 months, respectively.This study confirmed that the combined administration of IMRT and capecitabine can be used as an adjuvant therapy for gastric cancer patients, with few toxic side effects.

## Introduction

Gastric cancer is the fifth most common malignancy and the second leading cause of cancer death, worldwide, and this disease is especially prevalent in East Asia (1, 2). In the United States, 556 patients with R_0_ resection were enrolled in the Intergroup 0116 study. These patients were randomly assigned to a chemoradiotherapy (fluorouracil) group or a control group, and received expectant observation. The results confirmed that chemoradiotherapy had a positive effect for increasing the overall survival of patients at high risk for disease recurrence after surgery. The median overall survival times in the surgery only and chemoradiotherapy groups were 27 months and 36 months, respectively. The adjuvant treatment consisted of fluorouracil (425 mg/m^2^/day) plus leucovorin (20 mg/m^2^/day) for five days, followed by 4,500 cGy of radiation delivered as doses of 180 cGy per day (3). However, patient side effects in the Intergroup 0116 were significant. Grade 3 and grade 4 GI toxicities were found in 29% and 3% of patients, respectively. Furthermore, grade 3 and grade 4 hematologic toxicities were found in 26% and 28% of patients, respectively, and only 63% of these patients were able to complete their planned treatment regimen.

Capecitabine is an orally-administered chemotherapeutic agent used for treating metastatic breast and colorectal cancers. Furthermore, capecitabine is a prodrug that is enzymatically converted to 5-fluorouracil in the tumor, where it inhibits DNA synthesis and slows the growth of tumor tissue. The activation of capecitabine involves a systematic pathway that comprises three enzymatic steps and two intermediary metabolites, in which 5’-deoxy-5-fluorocytidine (5’-DFCR) and 5’-deoxy-5-fluorouridine (5’-DFUR) are combined to form 5-fluorouracil. Tham CK *et al.* reviewed a total of 108 patients, and found that patients in the radiotherapy (XRT) group achieved a similar median overall survival time (53 months) when compared to patients in the 5-Fu-RT group (54 months) (4). IMRT provides a more precise method of administering radiotherapy, in which full doses of radiation can be administered to areas at high risk for containing cancer cells, reducing the exposure received by high risk organs. This allows the physician to improve the disease control rate, while reducing toxicity (5-7). The exome array analysis identified the genetic variants that contributed to adverse reactions, and it was evidenced that the human leucocyte antigen (HLA) region was enriched for associations with Hand-and-Foot syndrome (*p *= 0.023). However, no specific single nucleotide polymorphisms (SNPs) or human leucocyte antigen (HLA) alleles were significant after Bonferroni correction (8). The present retrospective study of postoperative gastric cancer patients who subsequently received adjuvant therapy with combined IMRT and capecitabine was conducted to evaluate the clinical outcomes of these patients, as well as the toxicities associated with such treatment.

## Experimental

*Clinical Data*

A retrospective analysis of 31 patients (23 female and eight male patients; mean age: 57 years old) with pathologically confirmed gastric cancer, who were treated with surgery and adjuvant chemoradiotherapy between July 2009 and October 2011, was conducted. The inclusion criteria: the patients who received gastric cancer, pathological staging T3 or T4 or positive lymph node. The exclusion criteria: the patients who could not tolerate concurrent radiotherapy and chemotherapy. Two patients, who were originally included in the present study group, could not complete the radiotherapy due to toxicities, and were excluded in the final analysis. The tumor stage of all patents was confirmed according to guidelines in the 7^th^ edition of the American Joint Commission on Cancer (AJCC) Staging Manual, and all patients had an Eastern Cooperative Oncology Group performance grade of ≤2. Prior to treatment, each patient received an evaluation to rule out metastatic disease, and the chemoradiotherapy began following two cycles of postoperative chemotherapy. This study was approved by the Ethics Committee of Shanghai Jiao Tong University Affiliated Six People’s Hospital. Written informed consent was obtained from all the participants.

*Combined administration of intensity modulated radiotherapy (IMRT) and capecitabine*

All the patients underwent computed tomography (CT)-based treatment planning prior to receiving intensity modulated radiotherapy (IMRT). The target volumes and normal structures were contoured on a planning CT scan, and the clinical target volumes (CTVs) were calculated according to previously published guidelines, which included preoperative stomach volume, surgical bed including the stomach remnant, and perigastric lymph nodes (9, 10). Other lymph nodes such as mediastinal porta hepatis and peripancreatic nodes were included when these were considered to be at risk based on the location of the primary tumor, or pathologically involved lymph nodes. A contoured diagram of the bowel outside the planning treatment volume (PTV) was created, and the CTV to PTV expansion was typically 5-10 mm to compensate for daily setup error and organ motion. Normal structures such as the kidneys, liver, spinal cord, and bowel were also contour diagramed.

The IMRT plan was calculated by FOCUS. All the patients received 45 Gy/25 F for five times a week with beam energy of 6 MV. The following dose constraint guidelines were followed during the administration of IMRT: mean liver dose <20 Gy, 75% of the liver <15 Gy; 70% of each kidney <15 Gy or two-thirds of one kidney <18 Gy; 95% of the bowel <45 Gy; the spinal cord <45 Gy. The IMRT plan was normalized to 95% volume to administer 100% of the dose. The IMRT plan for each patient was designed to ensure optimal coverage while minimizing the dose received by normal structures. The dose volume histogram (DVH) was used to evaluate the radiotherapy plan. Acute toxicity was graded according to the RTOG Acute Radiation Morbidity Scoring Criteria (11). Dosing with capecitabine was initiated at one week following the initiation of radiotherapy, and was continued simultaneously with the radiotherapy for approximately 28 days. A gastric mucosa protector (*e.g.* proton pump inhibitor plus sucralfate) was used to ease the side effects of the radiotherapy (12). All the patients received follow-up examinations every 3-6 months by physical examination, magnetic resonance imaging (MRI), and assays for tumor markers. 

*Statistical Analysis*

 The Kaplan-Meier method was used to calculate the rates for locoregional control (LRC) and disease-free survival (DFS). Due to the short follow-up period used in the present study, the overall survival rates were not calculated. The time to a clinically meaningful event was calculated as the time elapsed after undergoing surgery.

## Results

The clinical and tumor characteristics of the 31 patients analyzed in the present study are illustrated in Table 1. The mean age of these patients was 57 ± 3.4 years old. Furthermore, among these patients, 30 patients (96.7%) had a performance status (PS) of 0 or 1, while one patient (3.3%) had a PS of 2. The stages of the disease were IIA in one patient (3.3%), IIB in five patients (16.1%), IIIA in eight patients (25.8%), IIIB in nine patients (29%), and IIIC in eight patients (25.8%). All the patients underwent D2 surgery, and the mean follow-up time was 33.0 months. 

Two patients, who were originally included in the study group, could not complete the radiotherapy due to severe gastro-intestinal toxicity, and were excluded from the statistical analysis. Two other patients had to temporarily discontinue the radiochemotherapy, but later continued treatment, while another 10 patients required a reduction in their dose of capecitabine. The acute toxicities recorded during the follow-up period are listed in Table 2. Although the most common adverse event was neutropenia in 15 patients (48.4%), no grade 3 or grade 4 neutropenic toxicities were reported. Gastro-intestinal toxicity was the second common adverse event, which occurred in 14 patients (45.2%), including two patients with GI grade 3 toxicity. 

Anemia and thrombocytopenia were the third most frequently reported adverse events, with seven cases (26%) each. The DVH data are presented in Table 3. The mean dose received by both kidneys was 1,439 ± 150 cGy, and the V20 for both kidneys was 24%. The mean dose delivered to the liver was 1,712 ± 180 cGy, and the V30 for the liver was 25.4%. The mean maximum dose delivered to the bowel was 4,808 ± 210 cGy, and also the V45 and V50 were 350 ± 15 cc and 25 ± 3 cc, respectively.

**Figure 1:**
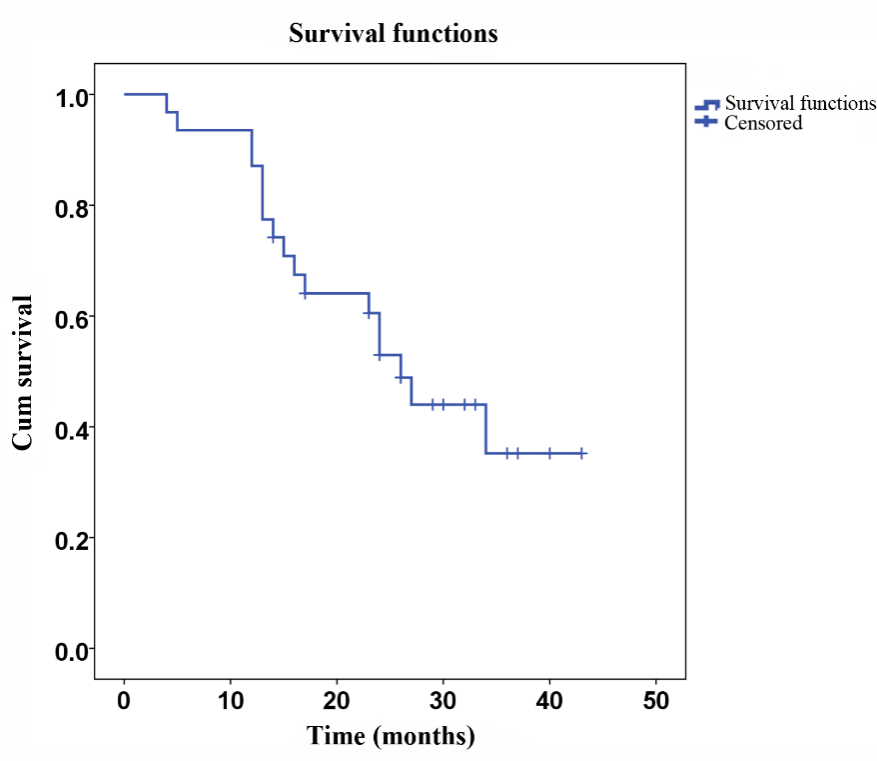
Disease free survival after surgery. Kaplan-Meier plot of DFS for 31 gastric cancer patients. The median DFS was 27.5 months

**Figure 2:**
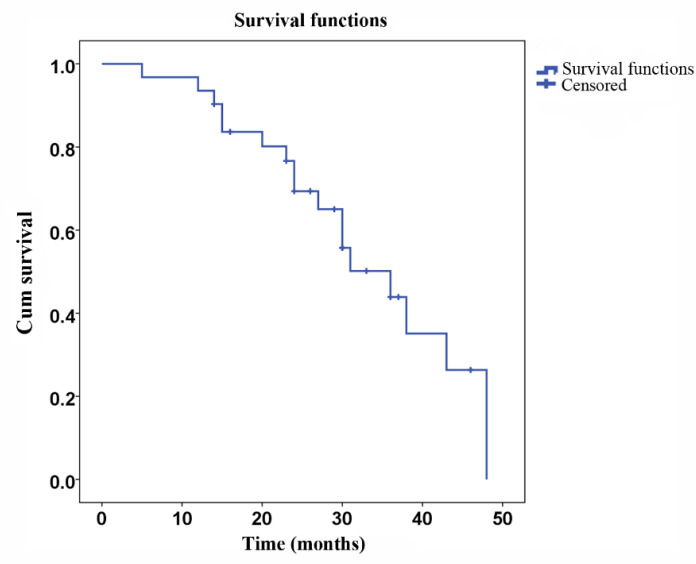
OS after surgical therapy. Kaplan-Meier plot of OS for 31 gastric cancer patients. The median OS was 32.9 months

A total of 31 patients were followed up for DFS and survival. The mean DFS was 27.5 months (SD ± 2.5 months, Figure 1), and the mean survival was 32.9 months (SD ± 2.6 months, Figure 2). Eighteen patients died during the follow-up period, among which eight deaths were due to relapse and 10 deaths were due to metastasis. No severe late occurring toxicities were reported during the follow-up periods.

## Discussion

Locoregional recurrence is a significant problem when treating gastric cancer, with reported rates ranging within 23%-38%. These rates suggest the need for effective adjuvant local therapy (13). The results of the Intergroup 0116 strongly suggested that chemoradiotherapy improves the local control rate of tumors, thereby improving the survival rate of gastric cancer patients. However, 17% of patients in that study did not complete their therapy due to toxic side effects, and 33% of patients experienced GI toxicity of ≥grade 3, which forced some of them to discontinue the chemoradiotherapy or reduce their dose of the drug. Such results support the need to improve therapy by reducing toxicity, while not reducing clinical efficacy. 

IMRT is a relatively complex form of radiotherapy that can be utilized to increase the radiation dose to the tumor and reduce the doses received by normal organs, thereby reducing the toxicity of chemoradiotherapy. In the present study, the mean dose received by the liver was 1,712 cGy, the mean bowel space that received >45 Gy was 350 cc, and the mean dose to the kidney was 1,429 cGy. All doses of radiation received by organs at risk (OARs) were less than the limiting dose. Hence, the side effects of radiotherapy were minimal. 

When treating gastrointestinal malignancies, the continuous infusion of 5-Fu has been preferred over 5-Fu bolos infusion (14, 15). In addition, oral capecitabine has been shown to be as therapeutically effective as the continuous infusion of 5-Fu (16, 17). The patients in the present study received oral capecitabine (800 mg/m^2^) starting from the fifth radiotherapy, and only two patients did not complete their radiotherapy regimen. In addition, the incidence and severity of side effects in the present study were less than those in the Intergroup 0116, while the clinical results were similar. Although the results of the Intergroup 0116 confirmed that chemoradiation administered following surgery produced better clinical results than surgery alone, the side effects experienced by the patients were severe, and 37% of these patients were not able to complete their planned treatment. Thus, clinical efficacy was greatly compromised. The patients in the present study received post-surgical adjuvant treatment with IMRT to reduce the dose of radiation received by the OARs. Furthermore, these patients were also treated with oral capecitabine, rather than 5-Fu, to reduce clinical side effects. As a result, only two patients (6%) did not complete the planned treatment regimen, and the incidence of severe side effects was greatly reduced. Although the treatment plans utilized in the present study followed the NCCN guidelines, the present results are limited by the absence of a control group, and it could not be concluded that the combined treatment of IMRT and capecitabine improves either PFS or overall survival. However, the present findings do confirm whether the combined treatment of IMRT and capecitabine can decrease the incidence and severity of the side effects. Although only 63% of patients in the Intergroup 0116 were able to endure their planned treatment regimen, the patients in the present study displayed good tolerance to their treatment. Some studies have shown that capecitabine had a similar safety profile, and may demonstrate superiority for PFS, when compared to 5-fluorouracil and cisplatin (FP), as a first-line treatment for Chinese patients (18, 19). In conclusion, the present study results show that the patients treated with IMRT and capecitabine tolerated their treatment well, and displayed few significant side effects. 

**Table 1:** Patients and tumor characteristics

**characteristics**		**N**	**Percent**
**Total**			
	Males	23	74.2
	Females	8	25.8
**Age**			
	Mean	57	
	< 50	8	25.8
	50-69	21	67.7
	>70	2	6.45
**PS**			
	0	24	77.4
	1	6	19.3
	2	1	3.3
**Tumor stage**			
	T_1_	1	3.3
	T_2_	2	6.4
	T_3_	16	51.6
	T_4_	12	38.7
**Lymph node stage**			
	N_0_	3	9.7
	N1	7	22.6
	N2	10	32.2
	N3	11	35.5
			

**Table 2:** Reported acute toxicities

**Adverse events**	**Grade 1**		**Grade 2**		**Grade 3**		**Grade 4**	
	**n**	**Percent**	**n**	**Percent**	**n**	**Percent**	**n**	**Percent**
Mucositis	0	0	0	0	0	0	0	0
GI	5	16.1	7	22.6	2	6.4	0	0
Neutropenia	5	16.1	10	32.2	0	0	0	0
Anemia	4	12.9	3	9.7	0	0	0	0
Thrombocytopenia	3	9.7	4	12.9	0	0	0	0
Dermatitis	0	0	0	0	0	0	0	0

**Table 3:** Dose volume histogram parameters of patients

**Organ**	**Mean**
Both kidneys cGy	1439
V20%	24
Liver cGy	1712
V30%	25.4
Bowel space	
V45 cc	350
V50 cc	25
Dmax Gy	4808
